# Acupoint Injection for Alleviating Side Effects of Chemotherapy in People with Cancer: A Systematic Review and Meta-Analysis

**DOI:** 10.1155/2021/9974315

**Published:** 2021-05-28

**Authors:** Yulan Yang, Hairong Su, Jian Wen, Jianyun Hong

**Affiliations:** ^1^Department of Acupuncture, Maoming People's Hospital, Maoming, Guangdong, China; ^2^Department of Oncology, Maoming People's Hospital, Maoming, Guangdong, China

## Abstract

**Objective:**

This study aimed to investigate the efficacy of acupoint injection for alleviating side effects of chemotherapy in people with cancer.

**Methods:**

PubMed, EMBASE, Cochrane library databases, CNKI, VIP, WanFang Date, and CBM were searched for randomized controlled trials (RCTs) from inception through December 28, 2020. This meta-analysis was performed using Review Manager 5.3.

**Results:**

A total of 8 RCTs including 557 participants were eligible and included in the meta-analysis. The selected RCTs studied acupoint injection for alleviating side effects of chemotherapy in people with cancer. Statistically significant improvements were observed for the incidence of nausea and vomiting (RR = 0.39; 95% CI 0.26, 0.58; *P* < 0.00001), the number of leukocyte (MD = 1.89; 95%CI 0.74, 3.03; *P* = 0.001), and the number of platelet (MD = 28.82; 95%CI 19.33, 38.30; *P* < 0.00001). Two of these studies suggested that acupoint injection can also reduce some other adverse reactions, which showed a statistical difference (RR = 0.29; 95% CI 0.11, 0.75; *P* = 0.01).

**Conclusion:**

The analysis indicated that acupoint injection can alleviate side effects of chemotherapy in people with cancer. However, due to the high risk of bias and small sample size in the included studies, the results need to be further confirmed by further large, rigorously designed trials.

## 1. Introduction

Cancer has already been a clinical difficult problem to threaten the health of mankind, because of its rising incidence rate and the aging of population. Fidler et al. [1] predict that over 20 million new cancer cases will be projected for 2025 in the world. Chemotherapy is one of the primary systemic adjuvant modality for cancer, which plays an important role in improving the survival for patients. However, the side effects of chemotherapy cannot be ignored, which seriously affect the treatment effect and quality of life of patients, and even termination of chemotherapy [2]. The common side effects include myelosuppression, digestive reaction (including nausea and vomiting, diarrhea, and anorexia), neurotoxicity, anaphylaxis, cytopenias (including leukopenia and neutropenia, thrombocytopenia, and anaemia), nephrotoxicity, hepatotoxicity, ototoxicity, cardiotoxicity, mucositis, stomatitis, pain, alopecia, cachexia, and asthenia [3]. Side effects are common in chemotherapy, persist throughout, and can be serious [4]. Although great progress has been made in managing the side effects of chemotherapy, these cannot satisfy the requirements of patients undergoing chemotherapy [5]. Thus, there is an urgent need to develop effective and safe alternative therapies.

Acupoint injection of traditional Chinese Medicine displays distinct clinical outcomes, and as a result, they have been increasingly used as an adjuvant therapy to manage side effects of chemotherapy.

## 2. Methods

### 2.1. Literature Search

The review was prepared according to the guidelines provided by the Preferred Reporting Items for Systematic Reviews and Meta-Analyses (PRISMA) statement [6] and the Cochrane Collaboration [7].

The following databases were searched for studies evaluating the efficacy of acupoint injection for alleviating side effects of chemotherapy in people with cancer from inception through December 28, 2020: PubMed, EMBASE, Cochrane library databases, the China National Knowledge Infrastructure (CNKI), Chinese Science and Technology Periodical Database (VIP), WanFang Data Information Site, and Chinese Biology Medicine Disc (CBMdisc).

The key search terms were “acupoint injection OR point injection OR acupuncture injection OR acupuncture point injection OR hydro-acupuncture”, “chemical therapy OR chemo therapy OR chemotherapeutic OR chemotherapy OR chemotherapy combined”, and “toxic reaction OR toxic response OR toxicity OR toxicity reaction OR side effects OR side reaction OR subsidiary reaction”. The detailed search strategy is shown in Table 1.

### 2.2. Study Selection

Inclusion criteria consisted of (1) randomized controlled trials (RCTs) in English or Chinese, (2) adult cancer patients diagnosed with any stage of cancer who suffered side effects of chemotherapy, and (3) the intervention of the experimental group as acupoint injection.

Exclusion criteria consisted of (1) no RCTs, (2) case reports, review articles, and animal experiments, and (3) side effects in people with cancer due to other causes.

**Figure 1:**
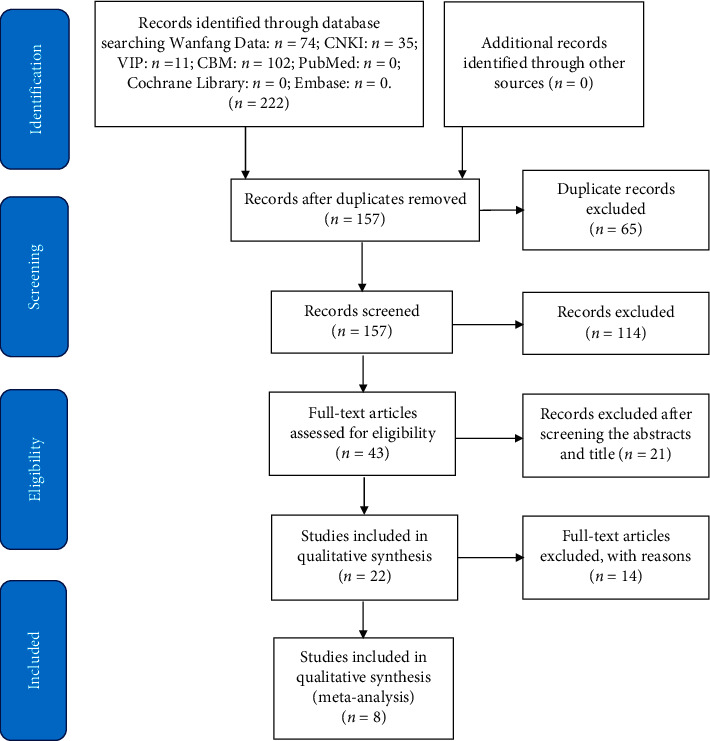
Flow chart of the study selection.

According to the specified search strategy, two authors (Yang and Su) identified 222 potentially articles and carried out the selection of research literature independently by importing into EndNote X9 software. The repetitive literatures were excluded first, and then the potentially eligible articles were identified after screening the titles and abstracts. Finally, we got a total of eight eligible studies for further analysis by going through the full text. The study selection process was shown in the PRISMA flow chart (Figure 1).

### 2.3. Data Extraction

The data of the studies included were extracted independently by two authors (Yang and Su), and disagreement was resolved by negotiation. The data included the primary author and year of publication, characteristics of patients, sample size, randomized method, interventions, inclusion and exclusion criteria, white blood cell count, platelet count, the incidence of nausea and vomiting, and other adverse events.

### 2.4. Bias Risk Assessment

To quantify the risk of bias, the Cochrane Handbook for Systematic Reviews of Interventions [8] was used to assess whether each study had a low, high, or unclear risk of bias. There were seven points that had to be independently evaluated by two reviewers: random allocation, allocation concealment, blinding of participants and personnel, blinding of outcome assessment, incomplete outcome data, selective outcome reporting and other bias. Disagreement was also resolved by discussion.

### 2.5. Data Synthesis and Analysis

The meta-analysis was performed using Review Manager software (RevMan, version 5.3), provided by the Cochrane library. Dichotomous and continuous data were analyzed using risk ratio (RR) and mean difference (MD) and standard mean differences (SMD), respectively, and the 95% confidence intervals (CIs) were computed for all outcomes. The potential heterogeneity across studies was assessed with a chi-squared test (Cochrane's Q statistic) and an I2 statistic. If substantial heterogeneity existed between studies, a random-effects model was utilized for data synthesis; otherwise, a fixed-effects model was chosen. When the continuous data among the studies were provided in the form of mean and range values, we used the method described by Hozo et al. [9] and Luo et al. [10] to calculate the standard deviations.

## 3. Results

### 3.1. Study Characteristics

The characteristics of each RCT are summarized in Table 2. A total of 8 RCTs [11–18] including 557 participants were eligible and included in the meta-analysis. All the studies were conducted in China, which sample sizes ranged from 40 to 96. In the control groups, 88 received oral or subcutaneous injection medications, 30 received conventional treatment and 140 received no further treatment. All patients in the treatment group received acupoint injection, and the acupoints were Zusanli (ST36), Xuehai (SP10), Neiguan (PC6), and Sanyinjiao (SP6).

### 3.2. Quality Assessment

**Figure 2:**
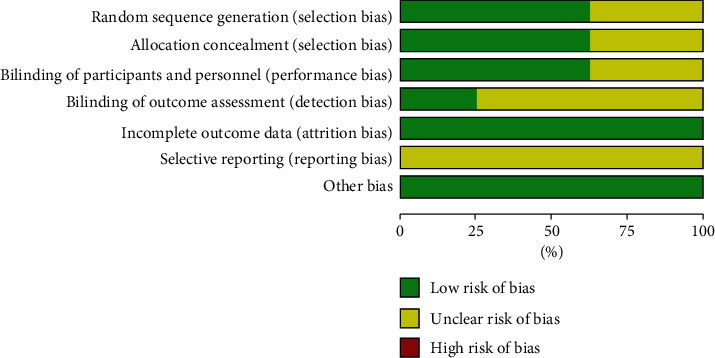
Risk of bias graph: review authors' judgements about each risk of bias item presented as percentages across all included studies.

**Figure 3:**
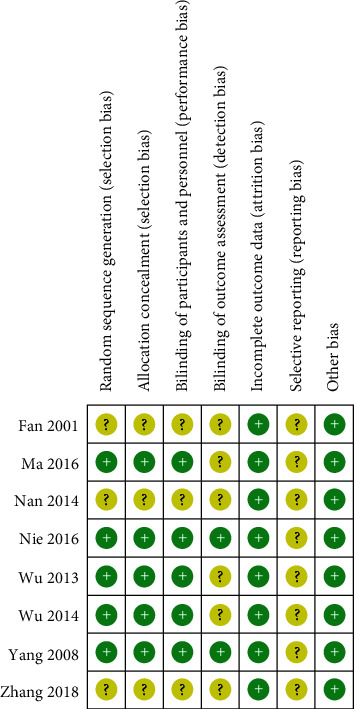
Risk of bias summary: review authors' judgements about each risk of bias item for each included study.

The quality assessment is summarized in Figures 2 and 3. Five trials [12, 14, 15, 17, 18] described the methods for random sequence generation as a random number table or random double blind method, and the others only mentioned that “random allocation” without detailed information. Five RCTs [12, 14, 15, 17, 18] were rated as low risk of bias, which reported allocation concealment. Two studies [17, 18] used the double blind method, three [12, 14, 15] used the single blind method, and the others did not adopt a blind method. All studies were assessed as low risk of bias because there were no missing data and all expected outcomes were reported. In all included trials, the risk of bias due to other reasons was identified as low because these studies appeared to be free of other sources of bias.

### 3.3. Result Analysis

#### 3.3.1. The Incidence of Nausea and Vomiting

**Figure 4:**
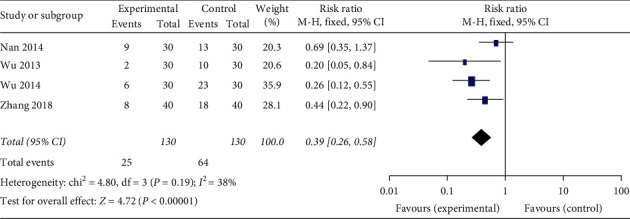
Forest plot of acupoint injection for alleviating the incidence of nausea and vomiting of chemotherapy in people with cancer.

Data pertaining to the incidence of nausea and vomiting after chemotherapy were analyzed in four included studies [13–16]. As no significant heterogeneity among the studies was detected (Chi^2^ = 4.80, *P* = 0.19; I^2^ = 38%; Figure 4), a fixed-effects model was applied to compare the efficacy of acupoint injection groups and control groups. Significant differences in the incidence of nausea and vomiting were detected between acupoint injection groups and control groups (RR = 0.39; 95%CI 0.26, 0.58; *P* < 0.00001).

#### 3.3.2. The Number of Leukocyte

**Figure 5:**
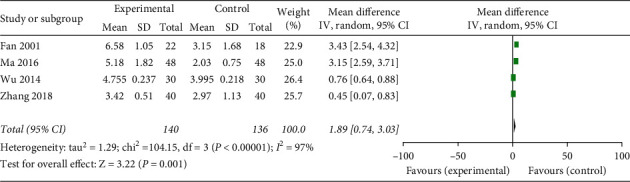
Forest plot of acupoint injection for the number of leukocyte of chemotherapy in people with cancer.

The data on effective rate of acupoint injection for the number of leukocyte after chemotherapy were provided in four included trials [11, 12, 14, 16]. As there was an evidence of significant heterogeneity between the trials (Chi^2^ = 104.15, *P* < 0.00001; *I*^2^ = 97%), the statistical analysis was performed using a random-effects model. Our meta-analysis demonstrated that acupoint injection was more effective for the number of leukocyte than western medications, conventional treatment, and no further treatment (MD = 1.89; 95%CI 0.74, 3.03; *P* = 0.001; Figure 5).

#### 3.3.3. The Number of Platelet

**Figure 6:**
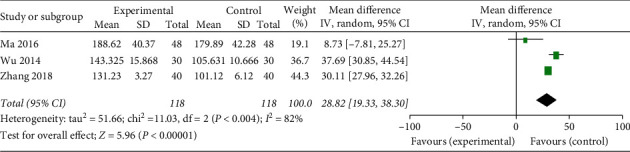
Forest plot of acupoint injection for the number of platelet of chemotherapy in people with cancer.

Of the eight included RCTs, three [12, 14, 16] provided data related to the number of platelet. A random-effects model was used for statistical analysis due to heterogeneity (Chi^2^ = 11.03, *P* = 0.004; *I*^2^ = 82%; Figure 6). Pooled analysis showed that the number of platelet was more greatly increased in the acupoint injection group than the other groups after chemotherapy (MD = 28.82; 95%CI 19.33, 38.30; *P* < 0.00001).

#### 3.3.4. The Incidence of Other Adverse Reactions

**Figure 7:**
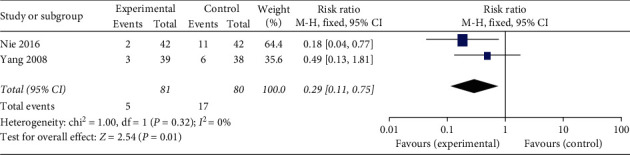
Forest plot of acupoint injection for the incidence of other adverse reactions of chemotherapy in people with cancer.

The incidence of other adverse reactions including thrombocytopenia, chills and fever, headache, fatigue, and muscle soreness was reported in two studies [17, 18]. The data available from the two studies using a fixed-effects model (Chi^2^ = 1.00, *P* = 0.32; I^2^ = 0%) demonstrated that acupoint injection can reduce the incidence of other adverse reactions in cancer patients after chemotherapy (RR = 0.29; 95% CI 0.11, 0.75; *P* = 0.01; Figure 7).

## 4. Discussion

The commonly used chemotherapy drugs have different toxicity profiles [19], which will produce a variety of side effects. The toxic and side effects of chemotherapy are involved in determining treatment choices, patient tolerability, and treatment success [20]. A study by Hsu et al. [21] assessed the incidence of side effects among chemotherapy in gynecological cancer patients and revealed that alleviating the side effects would be important to improve the quality of daily life and treatment willingness.

Acupuncture plays an important role in the treatment of many diseases. Acupoint injection is a common clinical therapy of acupuncture, and its role is widely known and recognized. Acupoint injection therapy is a method of injecting appropriate amount of liquid medicine into specific acupoints, combining the function of acupoints and the mechanical effect of acupuncture with the pharmacological effect of drugs to prevent and treat diseases. It has the characteristics of simple operation, safety, small dosage, rapid action, and easy acceptance by patients.

This study of eight studies including 557 participants aimed to investigate the efficacy of acupoint injection for alleviating side effects of chemotherapy in people with cancer. The results indicated that acupoint injection can alleviate side effects of chemotherapy in people with cancer.

The platinum agents (cisplatin, carboplatin, and oxaliplatin) are among the most useful chemotherapy drugs currently available to oncologists [22], and their common side effects included nausea and vomiting, bone marrow suppression, and renal toxicity neurotoxicity. Nausea and vomiting are the common and distressing side effects of chemotherapy from the perspective of cancer patients, so the treatment is generally far from satisfactory. In our meta-analysis, the incidence of nausea and vomiting data was pooled from four studies [13–16]. The result reflects a lower incidence of nausea and vomiting in the acupoint injection group (RR = 0.39; 95%CI 0.26, 0.58; *P* < 0.00001; Figure 4). It is also critical to emphasise prevention of delayed nausea and vomiting. However, none of the four trials reported the occurrence of delayed nausea and vomiting.

The most disconcerting side effect of cytotoxic chemotherapy is the bone marrow suppression, including leukopenia, neutropenia, thrombocytopenia, and anaemia [3, 23]. These side effects can cause dizziness, fatigue, and drowsiness and leave patients susceptible to infections and increase the length of their hospital stay. In our meta-analysis, the number of leukocyte data was also pooled from four studies [11, 12, 14, 16]. The result revealed that the acupoint injection was more effective for the number of leukocyte than western medications, conventional treatment, and no further treatment (MD = 1.89; 95%CI 0.74, 3.03; *P* = 0.001; Figure 5). And three RCTs [12, 14, 16] showed superior effects of acupoint injection on the number of platelet compared with no further treatment (MD = 28.82; 95%CI 19.33, 38.30; *P* < 0.00001; Figure 6).

Except for thrombocytopenia, two RCTs [17, 18] also reported the effects of acupoint injection on other adverse reactions, such as chills and fever, headache, fatigue, and muscle soreness. And there was a statistical difference on the incidence of these adverse reactions (RR = 0.29; 95% CI 0.11, 0.75; *P* = 0.01; Figure 7).

In addition, decreased appetite, altered taste, dizziness, constipation, hair loss, oral ulcers, peripheral neuropathy, etc., are also common major side effects of many chemotherapeutic agents in oncology. As there is no data retrieved, we did not conduct systematic analysis.

In terms of implications for clinical practice, the conclusion of our review provides some limited support for acupoint injection as a treatment for chemotherapy-related side effects. However, there remain many unsolved problems regarding acupoint injection treatment for alleviating side effects of chemotherapy in people with cancer, including selection of point prescriptions, selection of drugs, the number of sessions, or frequency of sessions.

Our meta-analysis has several limitations which must be considered. Firstly, all of the RCTs were conducted in China, which may cause publication bias. Secondly, the selection of point prescriptions, selection of drugs, the number of sessions, and frequency of sessions might increase the risk of bias. Thirdly, the flexibility of chemotherapy prescription could be a significant source of bias. Finally, the tumor type was inconsistently reported in the included RCTs, which could have a certain influence on our findings. Therefore, more rigorously designed RCTs conducted in different countries are warranted in the future.

## 5. Conclusions

Because of the effectiveness and safety of acupoint injection, our analysis supports that acupoint injection can be adopted as part of a multimodal approach for alleviating side effects of chemotherapy in people with cancer. To expand the application of acupoint injection on side effects of chemotherapy, more rigorous RCTs are required to investigate this matter.

## Figures and Tables

**Table 1:** Search strategy in Embase up till December 28, 2020 (similar search run in other databases).

1 “acupoint injection”:ti,ab,kw OR “'point injection”:ti,ab,kw OR “'acupuncture injection”:ti,ab,kw OR 'acupuncture point injection”:ti,ab,kw OR 'hydro acupuncture':ti,ab,kw
2 'chemical therapy':ti,ab,kw OR 'chemo therapy':ti,ab,kw OR chemotherapeutic:ti,ab,kw OR chemotherapy:ti,ab,kw OR 'chemotherapy combined':ti,ab,kw
3 'toxic reaction':ti,ab,kw OR 'toxic response':ti,ab,kw OR toxicity:ti,ab,kw OR 'toxicity reaction':ti,ab,kw OR 'side effects':ti,ab,kw OR 'side reaction':ti,ab,kw OR 'subsidiary reaction':ti,ab,kw
4 'randomized controlled trial':ti,ab,kw OR 'randomized':ti,ab,kw OR 'placebo':ti,ab,kw
5. #1 AND #2 AND #3 AND #4

**Table 2:** Characteristics of the included studies.

Author (year)	Diagnosis	Sample sizes (M/F)	Age (year)	Randomized method	Interventions	Acupoint selection of experimental group	Course of treatment	Outcome measures
Fan et al. (2001) [11]	Malignant tumor	E:22, C:18	Not reported	Not reported	E: hydroacupuncture with astragalus injection 4 ml, once a day, 6 days/session	Zusanli (ST36)	Three sessions	The number of leukocyte and immunoglobulin
					C: Batyl alcohol100 mg, leucogen 20 mg, three times a day, orally, 6 days/session	Sanyinjiao (SP6)		

Ma (2016) [12]	Malignant tumor	E:4 (27/21) C:48 (28/20)	E:48.23 ± 10.35	Random number table method	E: acupoint injection with Shenfu injection, once a day	Zusanli (ST36)	One day before chemotherapy, until the end of chemotherapy	The bone marrow suppression, changes of immune system and quality of life
			C:60.58 ± 10.73		C: no further treatment			

Nan (2014) [13]	Gastric cancer	E:30 (13/17)	E:63.8 ± 1.8	Not reported	E: acupoint injection with astragalus injection, once a day, 10 days/session	Zusanli (ST36)	From the day before chemotherapy to the day after chemotherapy	The bone marrow suppression, gastrointestinal toxicity
		C:30 (19/11)	C:63.3 ± 1.3		C: conventional treatment			

Wu et al. (2014) [14]	Tumor	E:30	Not reported	Random number table method	E: acupoint injection with astragalus injection, once every other day	Zusanli (ST36)	For 21 days	The incidence of nausea and vomiting, white blood cell and platelet count
		C:30			C: no further treatment			

Wu and Ji (2013) [15]	Lung cancer	E:30（20/10）	E:63.7 ± 5.2	Random number table method	E: acupoint injection of droperidol	Neiguan (PC6)	Not reported	The chemotherapy response, KPS score and quality of life
		C:30（19/11）	C:62.5 ± 5.4		C: no further treatment			

Zhang et al. (2018) [16]	Nonsmall cell lung cancer	E:40	Not reported	Not reported	E: acupoint injection with metoclopramide dihydrochloride injection 10 mg, once a day	Zusanli (ST36)	Not reported	The bone marrow suppression, gastrointestinal toxicity
		C:40			C: no further treatment			

Nie (2016) [17]	Malignant tumor	E:42（23/19）	Not reported	Random double blind method	E: acupoint injection with astragalus injection 2 ml/point, once a day, 7 days	Zusanli (ST36)	Not reported	Thrombocytopenia, chills and fever, headache, fatigue, muscle soreness
		C:42（20/22）			C: subcutaneous injection with recombinant human granulocyte colony-stimulating factor injection 150ug, once a day, 7 days	Xuehai (SP10)		

Yang (2008) [18]	Malignant tumor	E:39（20/19）	Not reported	Random double blind method	E: acupoint injection with dexamethasone 5 mg, once a day, 5 days	Zusanli (ST36)	Not reported	Thrombocytopenia, chills and fever, headache, fatigue, muscle soreness
		C:38（19/19）			C: subcutaneous injection with rhG-CSF, once a day, 3–5 days			
M, male; F, female; E, experimental group; C, control group; rhG-CSF, recombinant human granulocyte colony-stimulating factor								

## Data Availability

The data used to support the findings of this study are included within the article.
